# Modification of Fish Swarm Algorithm Based on Lévy Flight and Firefly Behavior

**DOI:** 10.1155/2018/9827372

**Published:** 2018-09-13

**Authors:** Zhenrui Peng, Kangli Dong, Hong Yin, Yu Bai

**Affiliations:** School of Mechatronic Engineering, Lanzhou Jiaotong University, Lanzhou, China

## Abstract

Artificial fish swarm algorithm easily converges to local optimum, especially in solving the global optimization problem of multidimensional and multiextreme value functions. To overcome this drawback, a novel fish swarm algorithm (LFFSA) based on Lévy flight and firefly behavior is proposed. LFFSA incorporates the moving strategy of firefly algorithm into two behavior patterns of fish swarm, i.e., chasing behavior and preying behavior. Furthermore, Lévy flight is introduced into the searching strategy. To limit the search band, nonlinear view and step size based on dynamic parameter are considered. Finally, the proposed algorithm LFFSA is validated with several benchmark problems. Numerical results demonstrate that LFFSA has a better performance in convergence speed and optimization accuracy than the other test algorithms.

## 1. Introduction

Optimization plays an important role in modern industry as well as in scientific world. Due to the computational costs of the existing numerical methods, researchers have to rely on metaheuristic algorithms to solve complex optimization problems.

Artificial fish swarm algorithm (AFSA) was proposed as a metaheuristic algorithm based on fish swarm behaviors—preying behavior, swarming behavior, and chasing behavior [1]. With its simple principle and such good features as robustness and tolerance of parameter setting, AFSA has become an increasingly important tool in swarm intelligence optimization. Some algorithms derived from AFSA have been presented: Wang et al. eliminated the step restriction and added new leaping behavior to improve the stability of the algorithm [2]. Another modified form of AFSA used particle swarm optimization to reformulate AFSA, integrated AFSA with communication behavior, and created formulas for major AFSA parameters [3]. Luan et al. adopted normal distribution function, Cauchy distribution function, multiparent crossover operator, mutation operator, and modified minimal generation gap model to overcome the drawback of slow convergence speed in later iterations [4]. Another improved AFSA with crossover and culture algorithm was proposed to enhance its optimization efficiency [5]. Hu et al. integrated the merits of the self-adaptation strategy, mutation strategy, and hybrid strategy into the social behaviors of AFSA [6].

Over the last decades, AFSA and its improved algorithms have been successfully applied to various engineering optimization problems. Kumar et al. adopted AFSA to optimize renewable energy sources in a microgrid [7]. By categorizing the social behaviors of fish swarm into foraging, reproductive, and random behaviors, a novel artificial fish swarm algorithm was advocated for solving large-scale reliability-redundancy application problem [8]. A derivative of AFSA was used to solve the multiobjective disassembly line balancing problem with fuzzy disassembly times [9]. A binary fish swarm algorithm was presented to solve profit-based unit commitment problem in generation companies [10].

Firefly algorithm (FA) is another metaheuristic algorithm based on the idealized behavior of the flashing characteristics of fireflies [11]. FA can adaptively adjust the radius of the induction and parallel search the optimum in multiple peaks. FA has natural advantages in solving multimodal optimization problem. Some drawbacks of searching strategy and parameters in FA were targeted and improved by researchers. A derivative of FA with directed movement of fireflies was proposed by Farahani et al. [12]. Chaotic sequence was introduced into the basic FA to adjust parameters *γ* and *α* by Coelho et al. [13]. Eagle Strategy, which combines Lévy flight search with FA, was introduced by Yang and Deb [14]. Elitist FA was presented [15], which tried to enhance the best solution position by generating *m* uniform random vectors and moving in the direction of best solution.

Firefly algorithm and its modified forms were also successfully applied to numerous practical problems. Jagatheesan et al. used firefly algorithm to design a controller for an automatic generation control of multiarea power thermal systems [16]. An FA-inspired band selection and optimized extreme learning machine were proposed for hyperspectral image classification [17]. A self-adaptive firefly algorithm was developed for placement of FACTS devices [18]. Teshome et al. modified FA to counteract some inherent problems that may hinder the performance of the maximum power point tracking [19]. Alb et al. used FA to solve a shielding/shunting electromagnetic problem [20]. Mishra et al. proposed a method for optimal placement of interline power flow controller by using FA [21]. Other applications of improved FA include image compression [22], financial forecasting [23], image segmentation [24], structural optimization [25], classification problem [26], unconstrained optimization [27], economic dispatch problems [28], clustering [29], image retrieval [30], and mechanical optimal design [31]. Some researchers presented comprehensive reviews of existing FA and its modified forms to encourage new researchers to employ FA for solving their own problems [32, 33].

At the same time, numerous studies have shown that Lévy flight is similar to flight characteristics of given animals and insects [34–36], which has been widely used in swarm intelligence algorithms. Subsequently, Lévy flight has been applied to optimization, and preliminary results showed its potential capabilities. Jensi and Jiji proposed an enhanced particle swarm optimization with Lévy flight [37]. Tang et al. proposed a new framework of shuffled frog-leaping algorithm based on the exploration and exploitation mechanism by using Lévy flight [38]. Yahya and Saka proposed a multiobjective artificial bee colony algorithm with Lévy flight and applied it to construction site layout planning [39].

Hybridization is recognized to be an important aspect of high performing algorithms in recent years [40]. Owing to some drawbacks of traditional AFSA and FA, they are not suitable for solving highly nonlinear and multimodal problems. By integrating the merits of AFSA with Lévy flight and FA, this paper proposes a novel hybrid algorithm, named LFFSA (fish swarm algorithm based on Lévy flight and firefly behavior), for global optimization. The highlights of the new algorithm are as follows:

- Attraction degree is involved in the definition of artificial fish
- Lévy flight is used to adjust the search route of artificial preying fishes
- By analyzing the relationship between swarming behavior and chasing behavior, unnecessary behavior (swarming behavior) is excluded instead of improving AFSA through adding new behaviors [3–6]
- Time complexity of the improved algorithm is also further analyzed to demonstrate the effectiveness of the improvement

The remainder of this paper is organized as follows: Section 2 describes the basic AFSA, FA, and Lévy flight respectively; Section 3 proposes and explains LFFSA algorithm in details; in Section 4, the superiority of proposed algorithm LFFSA is validated by several benchmark problems; Section 5 outlines the conclusion.

## 2. Background

### 2.1. Artificial Fish Swarm Algorithm

**Figure 1:**
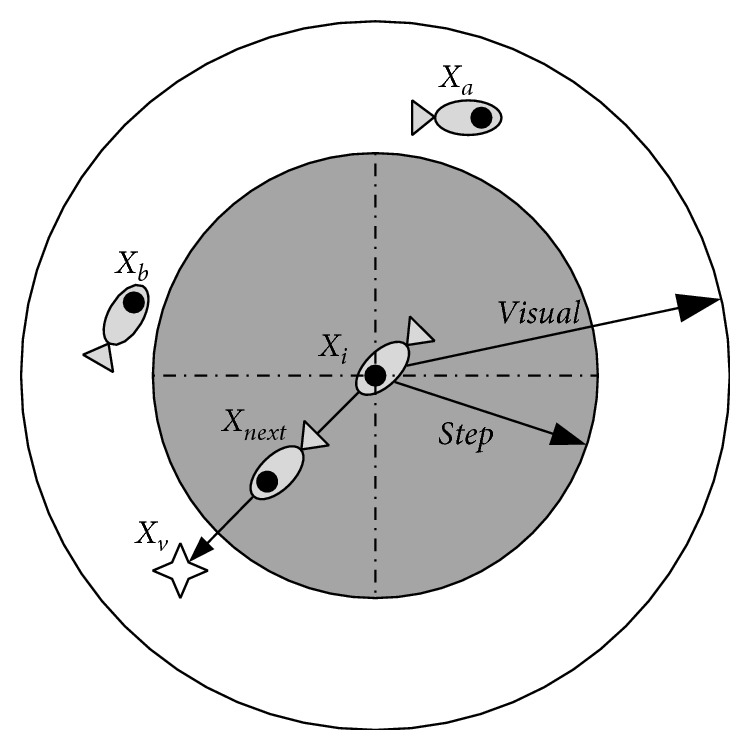
Vision concept of the artificial fish.

AFSA is a swarm intelligence algorithm, which can be employed to solve the optimization problem by imitating swarming, chasing, and preying behaviors of artificial fishes [1]. As shown in Figure 1, let *X*_*i*_ be the current position of one artificial fish, *X*_v_ be the viewpoint of artificial fish at one moment, Visual be the visual scope of each individual, *X*_a_ and *X*_b_ be fishes within the Visual of *X*_*i*_, Step be the biggest step of artificial fish, and *δ* be the congestion factor of fish swarm. The food concentration is proportional to the fitness function *f*(*X*). The behavior patterns of fish swarms can be described as follows.

*Swarming behavior*: if *f*(*X*_c_) > *f*(*X*_*i*_), where *X*_c_ is the central point inside the Visual of the point *X*_*i*_, swarming behavior is to be executed. Take *X*_c_ as *X*_v_. The fish at *X*_*i*_ will take a step toward the point *X*_c_.

*Chasing behavior*: if the point (denoted by *X*_max_) having the best objective function value inside the Visual satisfies *f*(*X*_max_) > *f*(*X*_*i*_), and if the Visual of *X*_*i*_ is not crowded, chasing behavior is to be executed. Take *X*_max_ as *X*_v_. The fish at *X*_*i*_ will take a step toward the point *X*_max_.

*Preying behavior*: preying behavior is tried in the following situations:

1. *f*(*X*_c_) < *f*(*X*_*i*_), *f*(*X*_max_) < *f*(*X*_*i*_), and the Visual is not crowded
2. The Visual is crowded

Here, a point *X*_*j*_ inside the Visual of *X*_*i*_ is randomly selected. If *f*(*X*_*j*_) > *f*(*X*_*i*_), the preying behavior is to be executed. Take *X*_*j*_ as *X*_v_. The fish at *X*_*i*_ will take a step toward the point *X*_*j*_. Otherwise, it will move a step randomly within its Visual.

The best solution obtained in each iteration is marked as “board.” After the specified iterations, search process is terminated and the result on the “board” is regarded as the final solution.

For artificial preying fishes, the position-updating can be formulated as

(1) $$\begin{array}{c} X_{\mathrm{next}}=X_{i}+\mathrm{rand}\cdot \frac{\text{step}\times \left(X_{j}-X_{i}\right)}{\mathrm{norm}\left(X_{j}-X_{i}\right)}\text{.} \end{array}$$

where *X*_next_ is the next position of artificial fish; *X*_*i*_ is the current position of artificial fish; *X*_*j*_ is the position which has a better objective function value; rand is a random number in [−1, 1]; and norm(*X*_*j*_ − *X*_*i*_) is the distance between two position vectors.

For artificial swarming fishes, the position-updating can be formulated as

(2) $$\begin{array}{c} X_{\mathrm{next}}=X_{i}+\mathrm{rand}\cdot \frac{\text{step}\times \left(X_{\text{c}}-X_{i}\right)}{\mathrm{norm}\left(X_{\text{c}}-X_{i}\right)}\text{.} \end{array}$$

For artificial chasing fishes, the position-updating can be formulated as

(3) $$\begin{array}{c} X_{\mathrm{next}}=X_{i}+\mathrm{rand}\cdot \frac{\text{step}\times \left(X_{\max}-X_{i}\right)}{\mathrm{norm}\left(X_{\max}-X_{i}\right)}\text{.} \end{array}$$

**Figure 2:**
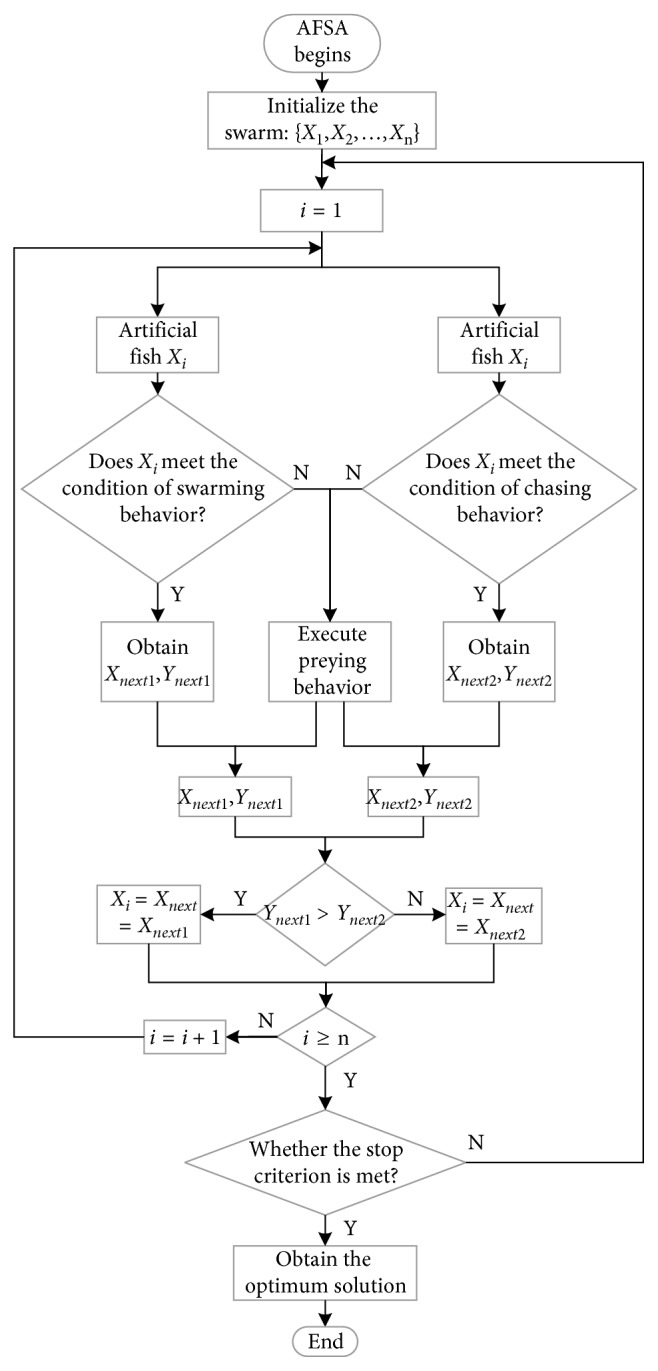
Flowchart of AFSA.

The flowchart of AFSA is shown in Figure 2.

### 2.2. Firefly Algorithm

Firefly algorithm (FA) [11] is another swarm intelligence algorithm. It achieves swarming phenomenon by using the fluorescent signal between two firefly individuals.

The attraction between fireflies depends on their light intensities and attraction degree. The light intensity is in proportion to the objective function value of firefly's position. Attraction degree is in proportion to the light intensity. The brighter the light intensity is, the higher attraction degree will be. Besides, the farther the distance is, the lower the light intensity and attraction degree will be.

In the simplest form, the light intensity *I*(*r*) varies with the distance *r* monotonically and exponentially as

(4) $$\begin{array}{c} I\left(r\right)=I_{0}\;\;{\text{e}}^{-\gamma r}\text{.} \end{array}$$

where *I*_0_ is the original light intensity and *γ* is the light absorption coefficient. As firefly attraction degree is proportional to the light intensity seen by adjacent fireflies, the attraction degree beta of a firefly can be defined as

(5) $$\begin{array}{c} \text{beta}={\text{beta}}_{0}\cdot {\text{e}}^{-\gamma r^{2}}, \end{array}$$

where beta_0_ is the attraction degree at *r*=0.

The distance *r*_*ij*_ between any two fireflies *i* and *j* at *x*_*i*_ and *x*_*j*_, respectively, is the Cartesian distance, which is calculated as

(6) $$\begin{array}{c} r_{ij}=\left\|x_{i}-x_{j}\right\|=\sqrt{\overset{n}{\underset{d=1}{\sum }}\left(x_{i\text{,}d}-x_{j\text{,}d}\right)}, \end{array}$$

where *n* is the dimensionality of the given problem.

### 2.3. Lévy Flight

Lévy flight is one kind of random searching strategy [35]. Flying step satisfies a heavy-tailed Lévy distribution, which can be represented by a clear power-law equation as

(7) $$\begin{array}{c} L\left(s\right)∼{\left|s\right|}^{1-\beta }, \end{array}$$

where *s* is random Lévy step. For searching problems inside a wide range of unknown space, the variance of Lévy movement increases faster than the dimensional Brownian movement.

To some extent, the foraging behavior of nature animals is a kind of random movement behavior. Because next movement usually depends on the current position and the probability of moving to next position, the effectiveness of each random movement becomes greatly important. Recent studies show that Lévy flight is one of the best searching strategies in random movement model [35, 41–43].

## 3. Fish Swarm Algorithm Based on Lévy Flight and Firefly Behavior

AFSA has several disadvantages in solving nonlinear and multimodal problems. Firstly, AFSA uses swarming behavior and chasing behavior to execute parallel search in a simple and fast way. However, after determining the direction, each artificial fish moving with random step will be unable to approach the target point effectively. Secondly, artificial fish will execute preying behavior when it does not meet the conditions of swarming behavior and chasing behavior. This kind of searching strategy is inefficient and can easily miss the optimum point. Thirdly, too many moving patterns can increase the algorithm complexity, which may cause slow convergence speed.

The above-listed drawbacks of AFSA are improved in the proposed LFFSA. FA has the unique moving strategy using attraction between fireflies, which can be used to fix the random moving after determining the direction in AFSA. And, the preying behavior can be improved using Lévy flight to specify the behavior of artificial fishes. In LFFSA, attraction degree is involved in the definition of artificial fishes, which allows each individual move according to attraction degree; Lévy flight is also considered in the definition of the artificial preying fish to avoid falling into the local optimum; chasing behavior is excluded to decrease the algorithm complexity.

**Figure 3:**
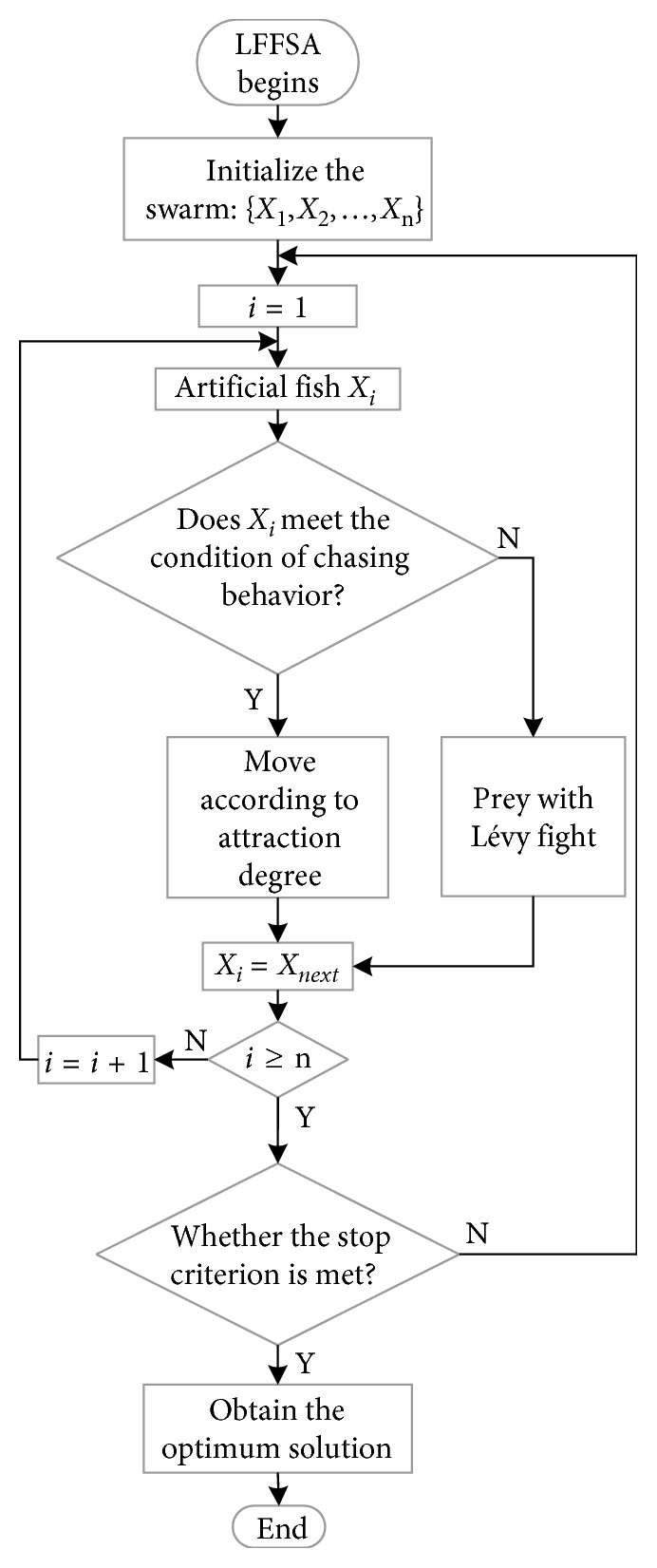
Flowchart of LFFSA.

**Figure 4:**
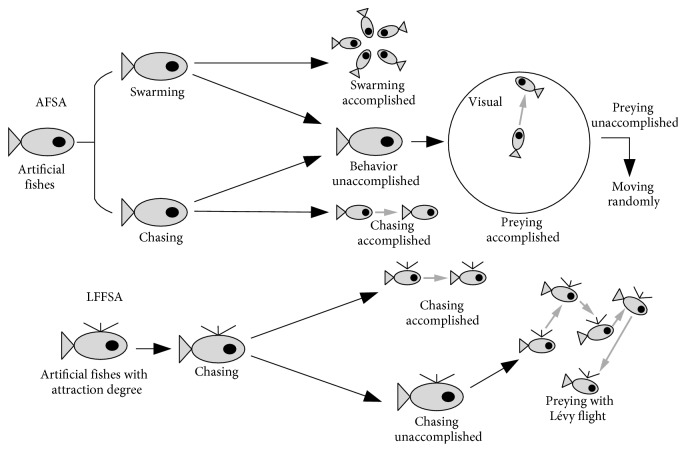
Mechanisms of AFSA and LFFSA.

The flowchart of LFFSA is shown in Figure 3. Pseudo code of LFFSA is described in Algorithm 1. To show the difference between AFSA and LFFSA vividly, mechanisms of both algorithms are provided in Figure 4.

The main improvements of LFFSA are summarized as follows:

(a) *Improvement 1*: FA-based moving strategy. Attraction degree is involved in the definition of artificial fishes, which can be formulated as
  (8) $$\begin{array}{c} {\text{beta}}_{ij}={\text{beta}}_{0}\cdot {\text{e}}^{-\gamma r_{ij}}, \end{array}$$
  where *r*_*ij*_ is the Cartesian distance between artificial fishes *i* and *j* given by Equation (6); *γ* is the light intensity coefficient, which can be set as a constant; and beta_0_, the largest attraction degree, is attraction degree of an artificial fish at *r*_*ij*_=0.

The position-updating with preying behavior established by attraction degree can be formulated as

(9) $$\begin{array}{c} X_{\mathrm{next}}=X_{i}+{\text{beta}}_{ij}\left(X_{j}-X_{i}\right)+\alpha \left(\mathrm{rand}-0.5\right), \end{array}$$

where *X*_*i*_ is the current position of artificial fish *i*; beta_*ij*_(*X*_*j*_ − *X*_*i*_) is the attraction degree; *α* is the step factor which is a constant between 0 and 1; rand is a number chosen randomly in [−1, 1]; and *α*(rand − 0.5) is to avoid falling into the local optimum.

(b) *Improvement 2*: inertia weight. A linear inertia weight is added into Equation (9) as
  (10) $$\begin{array}{c} X_{\mathrm{next}}=\omega_{t}X_{i}+{\text{beta}}_{ij}\left(X_{j}-X_{i}\right)+\alpha \left(\mathrm{rand}-0.5\right), \end{array}$$
  (11) $$\begin{array}{c} \omega_{t}=\omega_{\max}-\left(\omega_{\max}-\omega_{\min}\right)\cdot \text{gen}/\text{Maxgen}, \end{array}$$
  where *ω*_*t*_ is the weight size inherited from the last position of an artificial fish; *ω*_max_ denotes the biggest weight; *ω*_min_ is the minimum weight; gen represents the current iteration; and Maxgen is the ultimate iteration.

Similarly, the position-updating with chasing behavior can be formulated as

(12) $$\begin{array}{c} X_{\mathrm{next}}=\omega_{t}X_{i}+{\text{beta}}_{ij}\left(X_{\max}-X_{i}\right)+\alpha \left(\mathrm{rand}-0.5\right), \end{array}$$

where *X*_max_ is the position with the highest food concentration in the view of artificial fish *X*_*i*_ and other parameters are defined in Equations (10) and (11).

**Figure 5:**
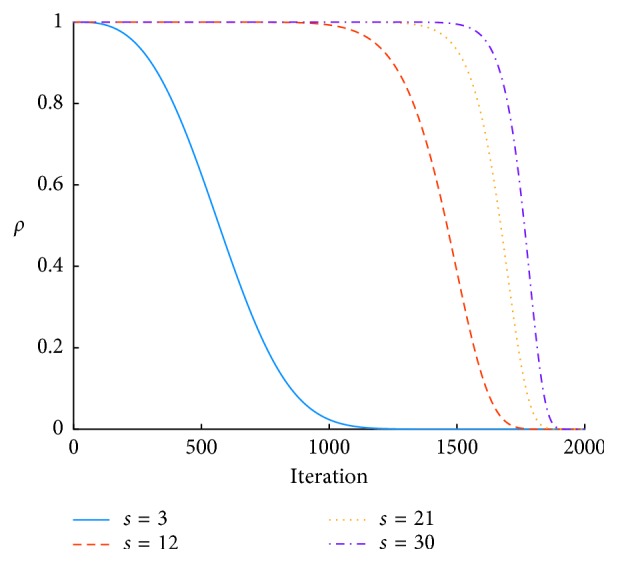
Value of *ρ*.

(c) *Improvement 3*: Lévy flight-based search strategy. Lévy flight is involved in the definition of artificial fishes are executing preying behavior. The movement can be formulated as
  (13) $$\begin{array}{c} X_{\mathrm{next}}=X_{i}+\alpha \;⊕\;L\left(\lambda \right), \end{array}$$
  (14) $$\begin{array}{c} L\left(\lambda \right)=\frac{\phi \times \mu }{{\left|v\right|}^{1/\beta }}\left(X_{i}-X_{\text{best}}\right), \end{array}$$
  where *X*_*i*_ is the current position of artificial fish *i*; ⊕ is the point to point multiplication; *L*(*λ*) denotes a random vector generated by Lévy flight; *X*_best_ represents the best fish on the “board”; *μ*=*t*^−*λ*^, 1 < *λ* < 3; and *μ* and *v* have the standard normal distribution *μ* ~ *N*(0, *ϕ*^2^), *v* ~ *N*(0,1), respectively, where
  (15) $$\begin{array}{c} \phi ={\left\{\frac{\Gamma \left(1+\beta \right)\sin\left(\pi \beta /2\right)}{\Gamma \left[\left(1+\beta \right)/2\right]\beta \cdot 2^{\left(\beta -1\right)/2}}\right\}}^{1/\beta }, \end{array}$$
  where Γ is the standard Gamma function.
(d) *Improvement 4*: nonlinear visual and step. Visual and step change nonlinearly and dynamically in LFFSA. The updating equations are as follows:
  (16) $$\begin{array}{c} \text{Visual}=\rho \cdot \text{Visual}+{\text{Visual}}_{\min}, \end{array}$$
  (17) $$\begin{array}{c} \text{Step}=\rho \cdot \text{Step}+{\text{Step}}_{\min}, \end{array}$$
  (18) $$\begin{array}{c} \rho =\exp\;\left(-30\times {\left(\frac{\text{gen}}{\text{Maxgen}}\right)}^{s}\right), \end{array}$$
  where Visual_min_, the minimum visual, takes 0.001; Step_min_, the minimum step, takes 0.0002; *ρ* is a nonlinear weight; and *s* represents an integer, *s* > 1. Here, *s*=3. Other parameters are the same as those in Equation (11). The relationship between *s* and *ρ* is as shown in Figure 5.

Besides, define the biggest distance between two artificial fishes as

(19) $$\begin{array}{c} \max D=\sqrt{{\left(x_{\max}-x_{\min}\right)}^{2}\times D}, \end{array}$$

where, *x*_max_ and *x*_min_ represent the upper bound and the lower bound of searching range respectively; *D* denotes the *D*-dimension searching space. The initial Visual is equal to max*D*, and initial Step is equal to max*D*/8. Then Visual and Step change dynamically according to Equations (16)–(18).

(e) *Improvement 5*: decrease of time complexity. Swarming behavior in AFSA is excluded to decrease the algorithm complexity.

## 4. Numerical Simulation

### 4.1. Comparison of Convergence Accuracy

LFFSA is validated by numerical simulations. Fish swarm algorithm with firefly behavior (FFSA), differential evolution (DE) algorithm, self-adaptive differential evolution (jDE) algorithm, and the two basic algorithms (AFSA and FA) are compared. All the algorithms are coded in Matlab 2014b. The operating system is windows 7. Simulation hardware is a PC with 2.50 GHz Inter Core i5 and 2.00 GB Memory.

Parameters shown in Table 1 are determined by trial and error.

**Figure 6:**
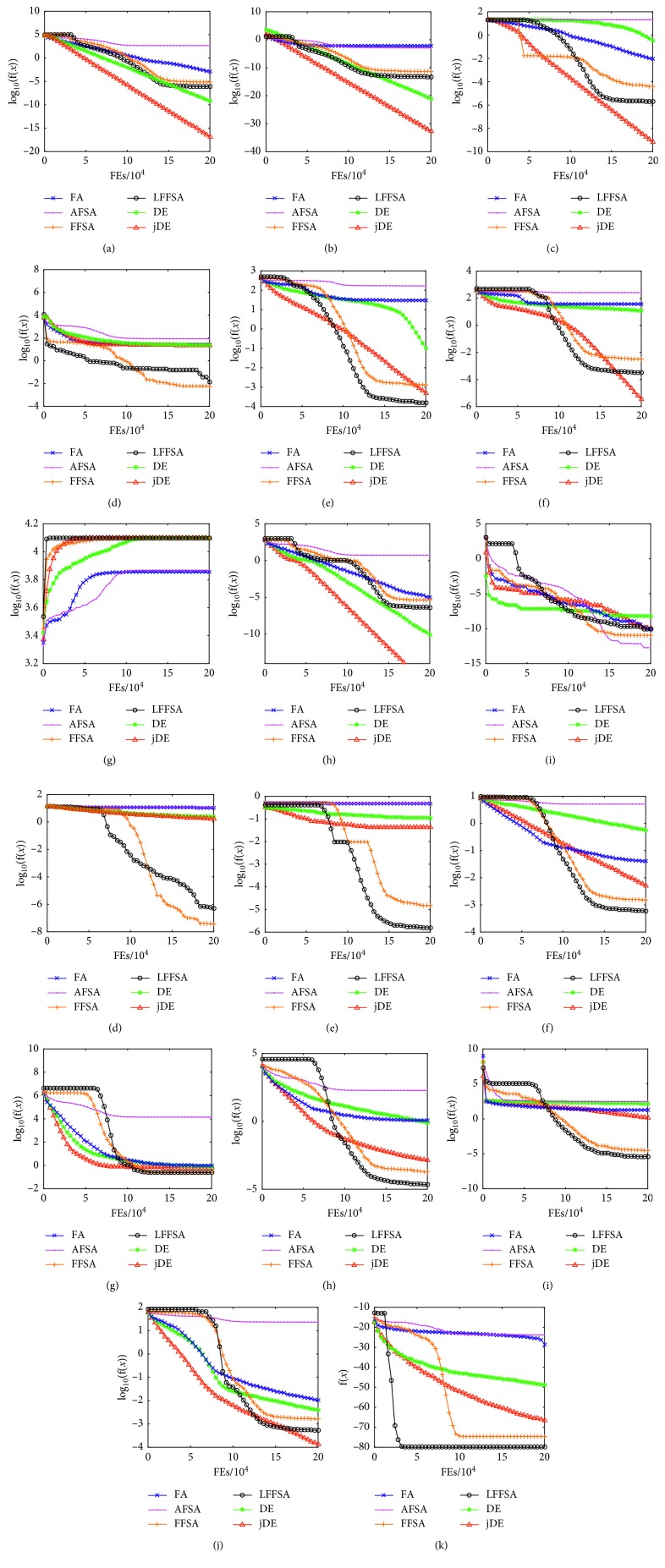
Iterative curves of test functions. (a) f1. (b) f2. (c) f3. (d) f4. (e) f5. (f) f6. (g) f7. (h) f8. (i) f9. (j) f10. (k) f11. (l) f12. (m) f13. (n) f14. (o) f15. (p) f16. (q) f7.

The following benchmarks are chosen carefully according to their features. Functions Sphere, Quartic, and Rosenbrock, etc., are simple unimodal problems. Functions Ackley, Rastrigin, and Schwefel, etc., are highly complex multimodal problems with many local minima. Schwefel function has a maximum value and other functions have minimum values. These benchmarks are listed in Table 2 [44, 45]. The solutions of 17 test functions obtained by different algorithms are compared. To compare the convergence speed and accuracy of the algorithms clearly and correctly, all functions are run 50 times for each algorithm, respectively. The results are averaged and plotted in Figure 6.

From Figure 6, LFFSA can avoid local optimum and have better convergence accuracy compared with the other algorithms. For AFSA and FA, the solutions of most functions are unsatisfactory; the DE cannot find ideal solutions of f3, f4, f5, f6, f10, f11, f12, f14, and f15; the jDE has good accuracy while solving some of those functions, e.g. f1, f2, f3, and f8; however, solutions of f4, f10, f11, and f15 obtained by jDE are not so precise; the LFFSA can obtain the ideal accuracy for almost all functions, although it cannot achieve a high precision level like solutions of f2 obtained by jDE; the FFSA is slightly worse than LFFSA. The LFFSA outperforms jDE in 10 benchmark functions, while 2 functions are comparative, and 5 functions are worse.

LFFSA is better than AFSA because Lévy flight is able to restrict the movement step of AFSA to a very small area around the current position. Furthermore, the attraction degree guides the fish moving. Besides, LFFSA can quickly lead the fish individual to the close-by optimal point. Considering all the advantages discussed above, the optimum solution can be found successfully by using LFFSA, which outperforms the basic algorithms for all test functions, and outperforms jDE for several functions. To observe the searching capabilities of different algorithms directly, the average, median, best, and worst values obtained by different algorithms are listed in Table 3. Results indicate that LFFSA can find ideal solutions and have a better robustness.

### 4.2. Computational Complexity Analysis

Time complexity is also an important indicator in the analysis of algorithms. If an algorithm is composed of several parts, then its complexity is the sum of the complexities of these parts. The algorithm may consist of a loop executed many times, and each time is with a different complexity. Time complexity of the algorithm is used to estimate the efficiency of the algorithm. It is defined that the time complexity of the algorithm, or the running time, is *O*(*f*(*n*)) [46]. Define *N* as the population.

In the definition of time complexity, *O*(*N*^2^) and *O*(*N*) are at different levels. If the time complexity of one algorithm is *O*(*N*^2^), the time complexity of the other one is *O*(*N*), then the former algorithm is more complex. In the other case, if the time complexity of one algorithm is *O*(*N*^2^) while the time complexity of the other one is *O*(*N*^2^+*N*), their complexities are both *O*(*N*^2^).

The time complexity analysis of AFSA is provided in Table 4.

From Table 4, the time complexity of AFSA is

(20) $$\begin{array}{c} O\left(\text{Maxgen }*\;\left(3\;*\;N^{2}+\text{trynumber }*\;N+6\;*\;N\right)\right)\text{.} \end{array}$$

Swarming behavior has *N* times of calculating congestion factor, 1 time of judging, and 1 time of moving. Therefore, time complexity of swarming behavior is *O*(*N*^2^+2 *∗* *N*). Chasing behavior has *N* times of calculating congestion factor, *N* times of searching, 1 time of judging, and 1 time of moving. Therefore, time complexity of chasing behavior is *O*(2 *∗* *N*^2^+2 *∗* *N*).

Time complexity analysis of LFFSA is listed in Table 5.

Due to the lack of swarming behavior, time complexity of LFFSA can be calculated as

(21) $$\begin{array}{c} O\left(\text{Maxgen }*\;\left(2\;*\;N^{2}+\text{Trynumber }*\;N+4\;*\;N\right)\right)\text{.} \end{array}$$

We can also obtain time complexity of FA:

(22) $$\begin{array}{c} O\left(\text{Maxgen }*\;\left(N^{2}+N\right)\right)\text{.} \end{array}$$

A conclusion can be obtained that time complexities of the three algorithms are at the same level. Their computational complexities in the worst case are only the square of the training sample size.

### 4.3. Experimental Complexity Analysis

Time complexity is a rough estimate of time cost. The more accurate time cost of an algorithm can only be validated by running it on computer. Since different algorithms cannot reach the same convergence accuracy, the test with fixed convergence accuracy is not available. Therefore, the test with max function evaluations is conducted. Running time of each algorithm is counted by the explorer of MATLAB. Parameter settings of algorithms are the same in Section 4.1. Average running time of different algorithms is listed in Table 6. When function evaluations are the same, running speed of LFFSA is faster than that of AFSA, while DE has the fastest running speed. Results are quite in accord with those obtained by computational complexity analysis. LFFSA and jDE are comparative in experimental complexity. Running time of FFSA is almost twice as much as that of LFFSA. The improvement of LFFSA decreases time complexity to some extent.

### 4.4. Parameter Analysis of LFFSA

**Figure 7:**
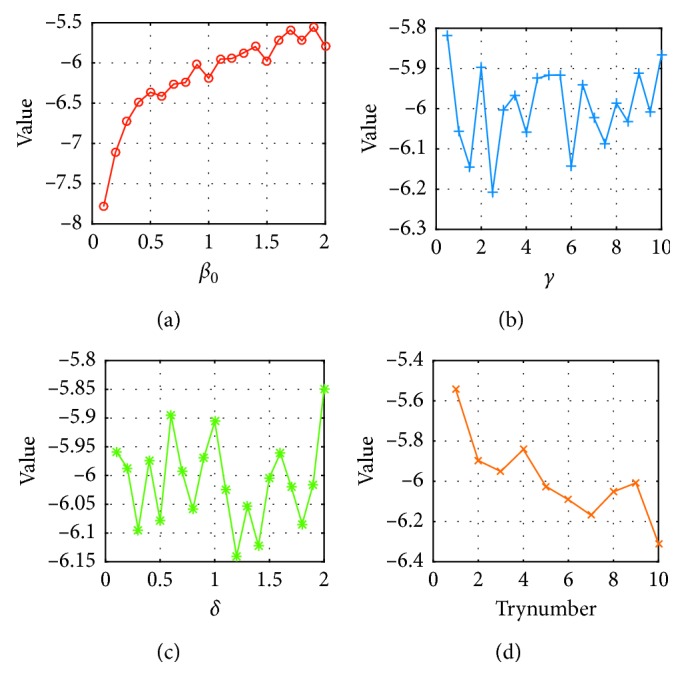
Test curves of parameters. (a) Test curve of *β*_0_. (b) Test curve of *γ*. (c) Test curve of *δ*. (d) Test curve of *Trynumber*.

The effect of parameters on optimization is analyzed in this section. Taking Ackley function as example, Figure 7 shows the change of the objective function value in the case of varying parameters. Trynumber and *β*_0_ are proportional to the optimization result. Trynumber can impact the time complexity of the algorithm, so the value should be appropriate not to affect the running speed. The best value of *γ* and *δ* is 2.5 and 1.2, respectively.

## 5. Conclusion

LFFSA is proposed to improve the capability of AFSA which integrates the merits of both AFSA and FA. Firstly, the searching characteristic of AFSA is studied by calculating the time complexity. Secondly, 17 benchmark test functions are used to verify LFFSA. Then time complexity of LFFSA is estimated. Numerical results demonstrate that LFFSA has a better performance in accuracy and speed of optimization to solve nonlinear optimization problems than the other test algorithms. However, the solution obtained by LFFSA can be more precise, and the way of modification could provide reference for those efficient algorithms, e.g. DE and GWO.

## Figures and Tables

**Algorithm 1:**
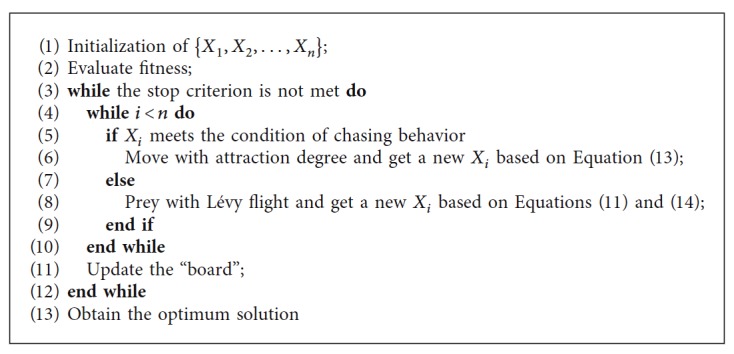
LFFSA.

**Table 1:** Parameter settings.

Algorithms	Parameters	Values
FA, FFSA, LFFSA	*β*_0_	1.0
FA, FFSA, LFFSA	*γ*	1.0
AFSA, FFSA, LFFSA	*δ*	0.618
AFSA, FFSA, LFFSA	Trynumber	5
DE, jDE	Scaling constant	0.5
DE, jDE	Crossover constant	0.9
All 6 algorithms	Population	50
All 6 algorithms	Maximum function evaluations (FEs)	2 × 10^5^

**Table 2:** Test functions.

No.	Test functions	Expression	Optimum value	Domain	*D*
f1	Sphere	*f*(*x*)=∑_*i*=1_^*D*^*x*_*i*_^2^	0	(−100,100)^*D*^	30
f2	Quartic	*f*(*x*)=∑_*i*=1_^*D*^*ix*_*i*_^4^	0	(−1.28, 1.28)^*D*^	30
f3	Ackley	$f\left(x\right)=-20\;\;\exp\left\{-0.2\sqrt{\left(1/D\right)\sum_{i=1}^{D}x_{i}^{2}}-\exp\left[\left(1/D\right)\sum_{i=1}^{D}\cos\left(2\pi x_{i}\right)\right]\right\}+20+e$	0	(−32.768, 32.768)^*D*^	30
f4	Rosenbrock	*f*(*x*)=∑_*i*=1_^*D*−1^100(*x*_*i*+1_ − *x*_*i*_^2^)^2^+(1 − *x*_*i*_)^2^	0	(−2.048, 2.048)^*D*^	30
f5	Rastrigin1	*f*(*x*)=∑_*i*=1_^*D*^{*x*_*i*_^2^ − 10cos(2*πx*_*i*_)+10}	0	(−5.12, 5.12)^*D*^	30
f6	Rastrigin2	*f*(*x*)=∑_*i*=1_^*D*^{*y*_*i*_^2^ − 10 cos(2*πy*_*i*_)+10}	0	(−5.12, 5.12)^*D*^	30
		$y_{i}=\left\{\begin{array}{cc} x_{i}, & \left|x_{i}\right|<\left(1/2\right),\\ \text{round}\left(2x_{i}\right)/2, & \left|x_{i}\right|>\left(1/2\right), \end{array}\right.$			
f7	Schwefel	$f\left(x\right)=\sum_{i=1}^{D}\left\{x_{i}\cdot \sin\;\;\sqrt{\left|x_{i}\right|}\right\}$	418.9829*D*	(−500,500)^*D*^	30
f8	Griewank	$f\left(x\right)=\left(1/4000\right)\sum_{i=1}^{D}x_{i}^{2}-\prod_{i=1}^{D}\cos\left(x_{i}/\sqrt{i}\right)+1$	0	(−600,600)^*D*^	30
f9	Quadric	*f*(*x*)=∑_*i*=1_^*D*^(∑_*j*=1_^*i*^*x*_*j*_)^2^	0	(−100,100)^*D*^	30
f10	Schaffer1	$f\left(x\right)=\sum_{i=1}^{D-1}\left\{\left(\left(\sin^{2}\;\;\sqrt{{x_{i+1}}^{2}+{x_{i}}^{2}}-0.5\right)/{\left(0.001\left({x_{i+1}}^{2}+{x_{i}}^{2}\right)+1\right)}^{2}\right)+0.5\right\}$	0	(−100,100)^*D*^	30
f11	Schaffer2	$f\left(x\right)=\sum_{i=1}^{D-1}\left\{\left(\sin^{2}\;\;\sqrt{\sum_{i=1}^{D}x_{i}^{2}}-0.5\right)/{\left(0.001\left(\sum_{i=1}^{D}x_{i}^{2}\right)+1\right)}^{2}+0.5\right\}$	0	(−100,100)^*D*^	30
f12	Maxmod	*f*(*x*)=max(|*x*_*i*_|)	0	(−10,10)^*D*^	30
f13	Dixon and price	(*x*_1_ − 1)^2^+∑_*i*=1_^*D*^*i*(2*x*_*i*_^2^ − *x*_*i*−1_)	0	(−10,10)^*D*^	30
f14	Powell	*f*(*x*)=∑_*i*=1_^*D*/4^[(*x*_4*i*−3_+10*x*_4*i*−2_)^2^+5(*x*_4*i*−1_ − *x*_4*i*_)^2^+(*x*_4*i*−2_ − 2*x*_4*i*−1_)^2^+10(*x*_4*i*−3_ − *x*_4*i*_)^4^]	0	(−4,5)^*D*^	28
f15	Zakharov	*f*(*x*)=∑_*i*=1_^*D*^*x*_*i*_^2^+(∑_*i*=1_^*D*^0.5*ix*_*i*_)^2^+(∑_*i*=1_^*D*^0.5*ix*_*i*_)^4^	0	(−5,10)^*D*^	30
f16	Sin1	∑_*i*=1_^*D*^|*x*_*i*_ sin(*x*_*i*_)+0.1*x*_*i*_|	0	(−10,10)^*D*^	30
f17	Sin2	*f*(*x*)=−∑_*i*=1_^*D*^sin(*x*_*i*_) sin^20^(*ix*_*i*_^2^/*π*)	−99.2784	(0, *π*)^*D*^	100

**Table 3:** Comparison of optimization results.

No.	Items	AFSA	Std.	FA	Std.	FFSA	Std.	LFFSA	Std.	DE	Std.	jDE	Std.
f1	Worst	3.014	0.181	−2.798	0.120	−5.132	0.071	−6.743	0.071	−8.544	0.132	−15.021	0.121
	Best	2.402		−3.257		−5.233		−8.500		−10.371		−17.242	
	Average	2.656		−3.048		−5.145		−7.278		−8.924		−16.326	
	Median	2.651		−3.102		−5.193		−6.325		−8.586		−16.706	

f2	Worst	−2.322	0.171	−1.9245	0.135	−11.178	0.074	−14.207	0.134	−20.506	0.400	−31.644	0.535
	Best	−4.126		−2.813		−11.313		−15.585		−21.611		−33.250	
	Average	−2.812		−2.278		−11.273		−14.812		−21.054		−32.687	
	Median	−2.562		−2.197		−11.271		−14.834		−20.970		−32.844	

f3	Worst	1.386	0.001	−1.776	0.124	−3.908	0.197	−6.121	0.138	−0.027	0.438	−8.934	0.167
	Best	1.307		−2.415		−4.647		−6.938		−1.281		−9.471	
	Average	1.587		−2.168		−4.147		−6.546		−0.049		−9.163	
	Median	1.586		−2.177		−4.225		−6.325		−0.035		−9.164	

f4	Worst	2.143	0.087	2.2733	0.097	−2.0348	0.125	−1.076	0.376	1.520	0.003	1.454	0.025
	Best	1.565		1.043		−2.416		−3.405		1.412		1.363	
	Average	1.946		1.476		−2.158		−1.946		1.385		1.312	
	Median	1.854		1.385		−2.235		−2.325		1.363		1.287	

f5	Worst	2.310	0.077	1.864	0.856	−2.846	0.044	−4.385	0.054	−0.579	0.323	0.898	6.009
	Best	2.096		1.243		−2.982		−5.145		−1.591		−12.831	
	Average	2.236		1.454		−2.946		−4.643		−0.999		−3.303	
	Median	2.136		1.285		−2.435		−4.325		−0.963		0.148	

f6	Worst	2.445	0.038	1.716	0.133	−2.382	0.076	−3.414	0.048	1.131	0.042	0.698	5.049
	Best	2.318		1.255		−2.618		−3.606		1.012		−11.404	
	Average	2.408		1.571		−2.486		−3.489		1.077		−5.466	
	Median	2.419		1.601		−2.462		−3.487		1.094		−7.632	

f7	Worst	3.815	0.018	3.7846	0.031	4.186	7.36*e* − 6	4.156	4.34*e* − 6	4.099	2.96*e* − 9	4.087	4.03*e* − 3
	Best	3.945		3.978		4.099		4.099		4.099		4.099	
	Average	3.813		3.848		4.099		4.099		4.099		4.097	
	Median	3.736		3.785		−4.099		−4.099		4.099		4.097	

f8	Worst	0.956	0.133	−0.960	0.223	−5.301	0.051	−6.271	0.055	−9.89	0.114	−Inf	0
	Best	−0.644		−0.500		−5.444		−6.455		−10.255		−Inf	
	Average	−0.735		−0.697		−5.357		−6.372		−10.071		−Inf	
	Median	0.736		0.685		−5.435		−6.325		−10.074		−Inf	

f9	Worst	−11.665	1.057	−9.76	1.324	−9.347	0.843	−8.695	1.323	−6.848	1.124	−6.131	2.697
	Best	−14.433		−10.574		−12.194		−11.937		−10.680		−14.831	
	Average	−12.786		−10.456		−10.764		−10.137		−8.178		−9.938	
	Median	1.136		1.085		−7.435		−6.325		−7.745		−9.663	

f10	Worst	0.957	0.131	0.974	0.223	−5.375	0.049	−6.274	0.056	0.673	0.032	0.280	0.110
	Best	0.644		0.497		−5.448		−6.486		0.483		−0.022	
	Average	0.747		0.649		−5.376		−6.348		0.526		0.230	
	Median	1.136		1.085		−7.435		−6.325		0.547		0.211	

f11	Worst	−0.301	2.72*e* − 05	−0.303	0.009	−4.751	0.071	−5.647	0.085	−0.896	0.103	−1.106	0.135
	Best	−0.301		−0.331		−5.011		−5.965		−1.107		−1.429	
	Average	−0.301		−0.313		−4.838		−5.804		−0.975		−1.364	
	Median	−0.301		−0.311		−4.835		−5.804		−0.896		−1.402	

f12	Worst	0.779	0.037	−1.177	0.163	−2.756	0.035	−3.158	0.037	−0.136	0.052	−1.904	0.221
	Best	0.658		−1.638		−2.892		−3.287		−0.339		−2.526	
	Average	0.711		−1.388		−2.811		−3.221		−0.252		−2.291	
	Median	0.716		−1.376		−2.801		−3.216		0.248		−2.374	

f13	Worst	4.286	0.173	0.574	0.297	−0.602	0.007	−0.602	0.005	0.039	0.068	−0.176	5.38*e* − 6
	Best	3.706		−0.175		−0.605		−0.605		−0.162		−0.176	
	Average	4.147		−0.013		−0.603		−0.603		−0.088		−0.176	
	Median	4.213		−0.135		−0.603		−6.603		−0.086		−0.176	

f14	Worst	2.404	0.084	0.405	0.318	−3.598	0.117	−4.514	0.101	0.276	0.211	−2.192	0.401
	Best	2.173		−0.576		−4.001		−4.869		−0.369		−3.409	
	Average	2.275		0.069		−3.773		−4.656		−0.117		−2.867	
	Median	2.246		0.133		−3.719		−4.651		−0.134		−2.924	

f15	Worst	2.588	0.0705	1.505	0.198	−4.281	0.117	−4.975	0.241	2.241	0.077	0.635	0.372
	Best	2.366		−4.679		−5.792		−7.2474		1.972		−0.370	
	Average	2.490		−4.561		−5.408		−6.3259		2.140		0.148	
	Median	2.506		−4.601		−5.456		−6.3259		2.136		0.218	

f16	Worst	1.438	0.058	−1.463	0.253	−2.762	0.026	−3.239	0.032	−2.354	0.042	−3.392	0.433
	Best	1.268		−2.296		−2.838		−3.334		−2.482		−4.646	
	Average	1.366		−1.987		−2.793		−3.282		−2.408		−3.868	
	Median	1.375		−2.016		−2.793		−3.284		−2.402		−3.751	

f17	Worst	−22.954	0.643	−25.673	1.721	−70.748	1.908	−79.645	0.131	−47.025	1.403	−63.031	1.942
	Best	−24.885		−32.075		−77.054		−80.098		−51.440		−68.738	
	Average	−23.772		−28.641		−74.598		−79.996		−48.982		−66.539	
	Median	−23.731		−28.759		−74.907		−80.001		−49.035		−66.559	

**Table 4:** Time complexity analysis of AFSA.

Procedure of AFSA	Time complexity
(1) Initialization of *N* artificial fishes	*O*(*N*)
(2) Initialization of ‘board'	*O*(*N*)
(3) Swarming behavior	*O*(*N*^2^+2 *∗* *N*)
(4) Chasing behavior	*O*(2 *∗* *N*^2^+2 *∗* *N*)
(5) Preying behavior	*O*(Trynumber *∗* *N*)
(6) Judging of terminal condition	*O*(1)
(7) Information output of ‘board'	*O*(1)

**Table 5:** Time complexity analysis of LFFSA.

Procedure of LFFSA	Time complexity
(1) Initialization of *N* artificial fishes	*O*(*N*)
(2) Initialization of ‘board'	*O*(*N*)
(3) Chasing behavior	*O*(2 *∗* *N*^2^+2 *∗* *N*)
(4) Preying behavior	*O*(trynumber *∗* *N*)
(5) Judging of terminal condition	*O*(1)
(6) Information output of ‘board'	*O*(1)

**Table 6:** Average running time of algorithms.

No.	Running time (s)					
	AFSA	FA	FFSA	LFFSA	DE	jDE
f1	10.23	7.47	18.5	7.53	4.64	6.08
f2	12.37	7.73	21.11	7.10	4.32	6.53
f3	10.87	7.80	20.73	8.96	4.12	8.25
f4	11.40	7.67	20.49	10.27	5.63	6.98
f5	10.53	7.66	19.47	8.03	3.28	5.96
f6	19.72	6.10	25.77	16.37	7.71	10.65
f7	10.53	7.43	18.77	8.56	3.27	6.56
f8	12.33	8.03	22.72	9.23	3.79	6.63
f9	20.83	7.20	21.36	7.71	4.01	6.84
f10	16.27	8.46	20.62	7.56	4.65	7.15
f11	9.26	8.95	13.74	6.35	3.63	6.67
f12	9.65	5.34	13.75	6.51	3.04	6.28
f13	9.12	5.26	14.36	10.41	4.13	7.15
f14	13.95	5.74	19.02	10.31	6.48	9.86
f15	9.45	5.39	13.07	7.36	4.62	7.74
f16	8.73	5.21	13.88	11.46	8.52	18.87
f17	14.53	7.28	18.41	5.96	3.32	6.44

## Data Availability

The data used to support the findings of this study are available from the corresponding author upon request.
